# Supplementary material for: Modeling glioblastoma heterogeneity as a dynamic network of cell states
Source: Mol Syst Biol. 2021 Sep 16;17(9):e10105. doi: 10.15252/msb.202010105 (PMC8444284; doi:10.15252/msb.202010105)
Supplement: Supplementary file 6 — Source Data for Figure 5 [file MSB-17-e10105-s004.zip › Figure5A_sourcedata/GSEA_3071/nosigdb_stateI.GseaPreranked.1622795145664/OLSEN_MESENCHYMAL.html]

Details for gene set OLSEN\_MESENCHYMAL[GSEA]

|  || Dataset | state13071 |
| Phenotype | NoPhenotypeAvailable |
| Upregulated in class | na\_pos |
| GeneSet | OLSEN\_MESENCHYMAL |
| Enrichment Score (ES) | 0.7609198 |
| Normalized Enrichment Score (NES) | 4.1749163 |
| Nominal p-value | 0.0 |
| FDR q-value | 0.0 |
| FWER p-Value | 0.0 |
Table: GSEA Results Summary

  

Fig 1: Enrichment plot: OLSEN\_MESENCHYMAL      
 Profile of the Running ES Score & Positions of GeneSet Members on the Rank Ordered List

  

| PROBE | GENE SYMBOL | GENE\_TITLE | RANK IN GENE LIST | RANK METRIC SCORE | RUNNING ES | CORE ENRICHMENT || 1 | MGP |  |  | 1 | 2.652 | 0.0370 | Yes |
| 2 | COL3A1 |  |  | 3 | 2.646 | 0.0739 | Yes |
| 3 | LUM |  |  | 4 | 2.203 | 0.1049 | Yes |
| 4 | DCN |  |  | 5 | 2.010 | 0.1332 | Yes |
| 5 | COL1A1 |  |  | 6 | 2.008 | 0.1614 | Yes |
| 6 | COL6A1 |  |  | 10 | 1.797 | 0.1858 | Yes |
| 7 | FN1 |  |  | 11 | 1.769 | 0.2107 | Yes |
| 8 | COL6A3 |  |  | 13 | 1.717 | 0.2346 | Yes |
| 9 | COL5A1 |  |  | 14 | 1.706 | 0.2586 | Yes |
| 10 | PCOLCE |  |  | 15 | 1.696 | 0.2824 | Yes |
| 11 | NUPR1 |  |  | 21 | 1.585 | 0.3032 | Yes |
| 12 | BGN |  |  | 24 | 1.568 | 0.3247 | Yes |
| 13 | COL4A1 |  |  | 28 | 1.524 | 0.3453 | Yes |
| 14 | POSTN |  |  | 32 | 1.494 | 0.3654 | Yes |
| 15 | IGFBP7 |  |  | 45 | 1.387 | 0.3814 | Yes |
| 16 | TIMP1 |  |  | 47 | 1.381 | 0.4006 | Yes |
| 17 | COL5A2 |  |  | 48 | 1.373 | 0.4199 | Yes |
| 18 | CTHRC1 |  |  | 49 | 1.370 | 0.4391 | Yes |
| 19 | COL6A2 |  |  | 67 | 1.237 | 0.4516 | Yes |
| 20 | SERPINF1 |  |  | 71 | 1.211 | 0.4677 | Yes |
| 21 | COL4A2 |  |  | 73 | 1.203 | 0.4844 | Yes |
| 22 | PPIC |  |  | 77 | 1.195 | 0.5003 | Yes |
| 23 | ID3 |  |  | 86 | 1.152 | 0.5142 | Yes |
| 24 | NNMT |  |  | 88 | 1.132 | 0.5298 | Yes |
| 25 | THY1 |  |  | 94 | 1.114 | 0.5440 | Yes |
| 26 | ZFP36L1 |  |  | 97 | 1.096 | 0.5589 | Yes |
| 27 | SPARC |  |  | 106 | 1.067 | 0.5716 | Yes |
| 28 | EMP1 |  |  | 122 | 1.012 | 0.5814 | Yes |
| 29 | NEAT1 |  |  | 123 | 0.982 | 0.5952 | Yes |
| 30 | NPC2 |  |  | 139 | 0.943 | 0.6041 | Yes |
| 31 | CST3 |  |  | 146 | 0.918 | 0.6153 | Yes |
| 32 | SERPINH1 |  |  | 157 | 0.902 | 0.6251 | Yes |
| 33 | VCAN |  |  | 197 | 0.833 | 0.6255 | Yes |
| 34 | IGFBP5 |  |  | 198 | 0.833 | 0.6372 | Yes |
| 35 | CALD1 |  |  | 201 | 0.824 | 0.6482 | Yes |
| 36 | S100A6 |  |  | 204 | 0.815 | 0.6591 | Yes |
| 37 | RRBP1 |  |  | 208 | 0.810 | 0.6696 | Yes |
| 38 | AEBP1 |  |  | 236 | 0.761 | 0.6725 | Yes |
| 39 | B2M |  |  | 241 | 0.744 | 0.6818 | Yes |
| 40 | LMNA |  |  | 247 | 0.739 | 0.6907 | Yes |
| 41 | PLAC9 |  |  | 254 | 0.731 | 0.6992 | Yes |
| 42 | IGFBP4 |  |  | 262 | 0.725 | 0.7074 | Yes |
| 43 | PRSS23 |  |  | 264 | 0.721 | 0.7172 | Yes |
| 44 | GSN |  |  | 265 | 0.720 | 0.7274 | Yes |
| 45 | IFITM3 |  |  | 268 | 0.714 | 0.7368 | Yes |
| 46 | S100A11 |  |  | 275 | 0.707 | 0.7450 | Yes |
| 47 | UACA |  |  | 296 | 0.679 | 0.7488 | Yes |
| 48 | S100A13 |  |  | 306 | 0.665 | 0.7555 | Yes |
| 49 | TPM2 |  |  | 320 | 0.655 | 0.7609 | Yes |
| 50 | MYL9 |  |  | 417 | 0.576 | 0.7411 | No |
| 51 | JUNB |  |  | 454 | 0.546 | 0.7383 | No |
| 52 | FSTL1 |  |  | 505 | 0.504 | 0.7309 | No |
| 53 | FTL |  |  | 564 | 0.473 | 0.7207 | No |
| 54 | CD63 |  |  | 618 | 0.444 | 0.7115 | No |
| 55 | EGR1 |  |  | 652 | 0.432 | 0.7080 | No |
| 56 | CD99 |  |  | 659 | 0.430 | 0.7123 | No |
| 57 | COL14A1 |  |  | 734 | 0.401 | 0.6964 | No |
| 58 | TIMP3 |  |  | 773 | 0.391 | 0.6909 | No |
| 59 | FOS |  |  | 832 | 0.370 | 0.6792 | No |
| 60 | IFITM2 |  |  | 836 | 0.370 | 0.6835 | No |
| 61 | FOSB |  |  | 854 | 0.364 | 0.6837 | No |
| 62 | TPT1 |  |  | 971 | 0.334 | 0.6547 | No |
| 63 | LY6E |  |  | 1030 | 0.321 | 0.6424 | No |
| 64 | IGFBP6 |  |  | 1048 | 0.318 | 0.6419 | No |
| 65 | VIM |  |  | 1105 | 0.305 | 0.6299 | No |
| 66 | SELENOM |  |  | 1365 | 0.258 | 0.5582 | No |
| 67 | RPL41 |  |  | 1600 | -0.267 | 0.4940 | No |
| 68 | EEF1D |  |  | 1769 | -0.290 | 0.4492 | No |
| 69 | RPL32 |  |  | 2741 | -0.483 | 0.1737 | No |
| 70 | MT2A |  |  | 2910 | -0.547 | 0.1326 | No |
| 71 | LIMA1 |  |  | 3429 | -1.353 | 0.0010 | No |
| 72 | LHFPL6 |  |  | 3453 | -1.600 | 0.0169 | No |
Table: GSEA details [plain text format]

  

Fig 2: OLSEN\_MESENCHYMAL: Random ES distribution      
 Gene set null distribution of ES for **OLSEN\_MESENCHYMAL**
